# Effects of COVID-19 on the laboratory turn-around time of vaccine-preventable disease surveillance: the case of measles in South Sudan

**DOI:** 10.11604/pamj.2020.37.245.24506

**Published:** 2020-11-17

**Authors:** David Majuch Kunjok, Isaac Michael Zingbondo

**Affiliations:** 1 African Field Epidemiology Network (AFENET), Juba, South Sudan,; 2 Ministry of Health, Juba, South Sudan

**Keywords:** South Sudan, laboratory, time turnaround, measles

## To the editors of the Pan African Medical Journal

Corona Virus Disease (COVID-19) is caused by severe acute respiratory coronavirus type 2 (SARS-CoV-2), which was first reported in Wuhan, China [1,2]. The transmission of the virus spread to many different countries, which compelled the World Health Organization (WHO) to declare the virus as a pandemic in March 2020 [3,4]. The first case of the Coronavirus was detected in South Sudan in the middle of March, prompting restriction of inter-States movement. The national public health laboratory performing tests, to detect Vacine-preventable Disease (VPDs) are also responsible for conducting SARS-CoV-2 tests, which is straining its capacity. In addition, disruption of the transportation of specimens to the national laboratory from the ten States of South Sudan has led to the long lead time. We used the 2019 and 2020 VPDs data sources from the South Sudan ministry of health to assess the impact of COVID-19 on the measles laboratory turn-around time in South Sudan.

Vaccine-Preventable Disease surveillance activities are affected by COVID-19 pandemic control measures, including the partial lockdown; hence, to detect measles during COVID-19, South Sudan must maintain or develop contingency measures to identify cases, although potentially at reduced levels, with a decreased frequency.

### Laboratory turnaround time for Measles

The performance of the National Public Health laboratory (Table 1) in terms of work efficiency on turn-around time (time from when the specimens are sent, and the time the laboratory results are sent back to the field). Comparing the same time in 2019, there is a slight fall back in the performance. This is attributed to the COVID-19 pandemic since the measles laboratory is already overwhelmed by COVID-19 activities. In addition to that, the lockdown and restrictions on inter-State movements have negatively impacted on the surveillance activities as specimens took long to be sent to the next levels. In addition to that, shortages in the reagents and workload have also contributed to the decline in laboratory performance. Rapid feedback to the lower levels (States and counties) is crucial for timely interventions such as measles reactive campaigns [5,6].

**Table 1:** summary of national public health laboratory performance, Jan-June 2020, South Sudan

Month	Average lead time 2019	Average lead time 2020
Jan	8	13
Feb	10	14
Mar	8	12
Apr	9	16
May	7	0
Jun	8	33
Jul	14	
Aug	12	
Sep	11	
Oct	14	
Nov	8	
Dec	7	

**Figure 1:**
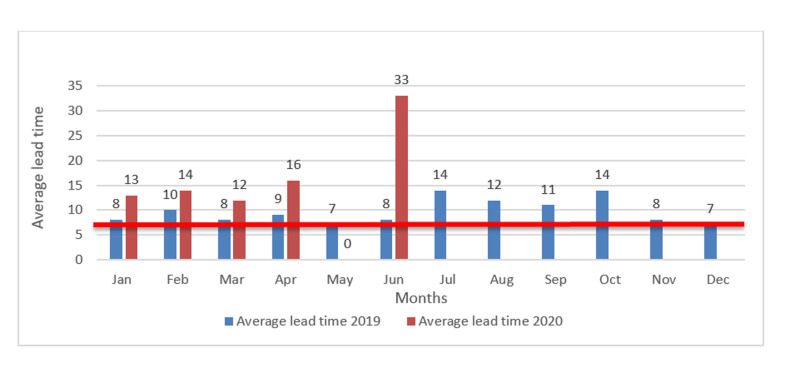
comparison of average turnaround time for measles laboratory performance 2019/2020, South Sudan

All results should be within a week of receipt of the serum sample and positive cases should be reported within 24 hours [7]. The laboratory performance seems to shift away from the expectation as specimens take long to be examined and send feedback to the field (Figure 1). The month of June is experiencing the highest turn around as specimens are still pending for an average of 32 days from the time received at the national laboratory to the date of releasing laboratory results. Turn-around time can be affected by many factors, which include: timing of specimen collection and transportation of specimens to laboratories. The standard turn-around time is considered to be 1-5 days for serology [8]. The information from the laboratory is useful for monitoring laboratory performance and feedback to the States for timely intervention [8]. However, COVID-19 has made it impossible to have timely reports for measles reactive intervention. The Non-measles Febrile Rash Illness rate (NMFRI) implies the sensitivity of the surveillance in detecting suspected measles cases in the country. There is a significant decline (Table 2) in the surveillance performance both in detecting suspected measles cases and investigating the case so adequately in 2020 compared to the previous years. This is majorly attributed to the COVID-19 pandemic, restricting the surveillance movements to the health facilities and communities. The investigation of sporadic cases of measles and chains of transmission has become increasingly difficult during the time of the COVID-19 pandemic [5]. The current situation has also changed community perception in seeking health services.

**Table 2:** non measles febrile rash illness rates adequacy of investigation from 2015-2020

State of Residence	NMFRI Rate 2015	NMFRI Rate 2016	NMFRI Rate 2017	NMFRI Rate 2018	NMFRI Rate 2019	NMFRI Rate 2020	Investigation Rate 2020
CENTRAL EQUATORIA	3.1	3.0	2.9	2.8	2.7	2.6	100.0
EASTERN EQUATORIA	2.7	2.6	2.6	2.5	1.7	1.1	26.1
JONGLEI	0.3	0.7	1.5	2.0	1.9	0.8	100.0
LAKES	0.9	0.5	1.2	1.9	1.8	0.8	100.0
NBG	0.2	1.6	2.8	0.9	3.2	0.2	98.9
UNITY	1.8	1.8	1.7	1.8	1.9	1.6	100.0
UPPER NILE	0.9	0.9	0.9	0.9	2.8	0.8	0.0
WARRAP	0.1	0.4	0.1	0.4	1.8	0.1	100.0
WBG	4.1	4.0	3.9	3.8	3.9	3.5	95.7
WEQ	3.6	3.5	3.4	3.3	2.1	2.0	88.7
**South Sudan**	**1.5**	**1.5**	**1.4**	**1.4**	**1.1**	**1.1**	**80.9**

The average adequate investigation of measles cases at the national in the year 2020 is 80.9 (Table 2). This is much far below 95% in 2019 when compared, while the NMFRI rate has shown the downward trends across the States of South Sudan. The incidence of measles cases was anticipated to decline by the end 2020, while a decline in routine vaccine uptake due to COVID-19 will increase the incidence and surpass the recent years due to the increase in the number of susceptible children. The outbreaks of VPDs may surge if the lockdown ends and social distancing are relaxed and schools reopen [9].

In diseases earmarked for elimination such as measles, every single case must be identified and investigated. Timely detection, reporting, and feedback are required to support rapid public health interventions. The role of surveillance systems is to ensure that adequate samples are collected correctly and on time during infection. With the interruption of surveillance systems by COVID-19, measles cases are expected to increase by 2020/2021, if there are no adequate supplementary measures put in place.
